# Gestation Regulates Growth Hormone and Its Receptor Expression in Sheep Immune Organs

**DOI:** 10.3390/biology14101318

**Published:** 2025-09-24

**Authors:** Zhouyuan Li, Xiaoxin Ma, Ziwang Du, Jingjing Li, Leying Zhang, Ling Yang

**Affiliations:** School of Life Sciences and Food Engineering, Hebei University of Engineering, Handan 056038, China; 13213533399@163.com (Z.L.); 18632453165@163.com (X.M.); dzw15831043254@163.com (Z.D.); 15137164507@163.com (J.L.); zhangly056000@126.com (L.Z.)

**Keywords:** growth hormone, growth hormone receptor, gestation, ewe

## Abstract

There are multiple adaptations in maternal physiology, hormones, and immunology during pregnancy. Growth hormone (GH) is not only produced by the pituitary but also secreted by extra-pituitary tissues. In this study, 24 ewes were randomly divided into four groups and mated with either adult intact rams (pregnant ewes) or a vasectomized ram (nonpregnant ewes), and maternal thymus, lymph node, spleen, and liver were harvested at day 16 of nongestation and at days 13, 16, and 25 of gestation. The specified primers for *GH* and GH receptor (*GHR*) were utilized to analyze mRNA expression of *GH* and *GHR* using quantitative real-time PCR. Specified anti-GH1 antibody and anti-GHR antibody were used to detect protein expression of GH and GHR using Western blot and immunohistochemical analysis. The results revealed that there were increases in GH and GHR in the maternal spleen, GH in the liver, and GHR in the thymus and lymph nodes, but a downregulation of GH in lymph nodes during early gestation. In addition, early gestation affected GH expression in the thymus and GHR in the liver. In conclusion, it is reported for the first time that early gestation modulates GH and GHR expression in these maternal organs in a tissue-specific manner, which may regulate the function of these immune organs in ewes.

## Simple Summary

Pregnancy induces adaptations in maternal hormones and immunology, and growth hormone (GH) can be produced by the pituitary and extra-pituitary tissues. This research revealed that gestation regulated GH and its receptor expression in the maternal immune organs, including the thymus, lymph node, spleen, and liver, in a tissue-specific manner, which is associated with adaptations in these maternal organs during early gestation in sheep.

## 1. Introduction

Growth hormone (GH) is secreted by the pituitary somatotrophs and acts on multiple cell types, tissues, and organs, which play key roles in growth and metabolism [1]. GH produced by cells of the immune system is similar to that secreted by the pituitary, and lymphocyte GH has an autocrine/paracrine effect on the spleen and thymus in mice [2]. GH can enhance thymic secretion of cytokines and thymic hormones and also improves thymic epithelial cell proliferation and thymocyte proliferation and migration [3]. GH is involved in trafficking naive CD4^+^CD8^−^ cells to the peripheral lymph nodes, which is mediated by the chemokine CXCL12 [4]. GH has effects on the activity of calcineurin, which is implicated in T cell activation and gluconeogenesis, and directly via the GH receptor (GHR) in rat liver [5]. Pituitary GH release and expression of hepatic GHR are related to estradiol concentrations during the estrous cycle in the cows [6]. Therefore, GH exerts its effects on immune organs via GHR.

There is a hormonal and immunological adaptation in females during normal gestation, which results in a complexity and unique circumstance in the immune system [7]. There are differences between pregnant and nonpregnant ewes in the immune functions of the lymph nodes, which are associated with changes in the female immune system [8]. As a central lymphoid organ, the thymus changes markedly during pregnancy, which is associated with Treg cell development [9]. The splenic antigen-presenting cells express differential costimulatory molecules during gestation, which are associated with the tolerogenic immune response in mice [10]. Maternal liver size and function are modulated by pregnancy, which are related to the accommodation of dramatic changes in metabolic demands during pregnancy in humans [11]. It is known that two genetically different individuals coexist during pregnancy in mammals, but it remains controversial that the maternal immune system plays roles in reproductive success in humans [12].

**Figure 1:**
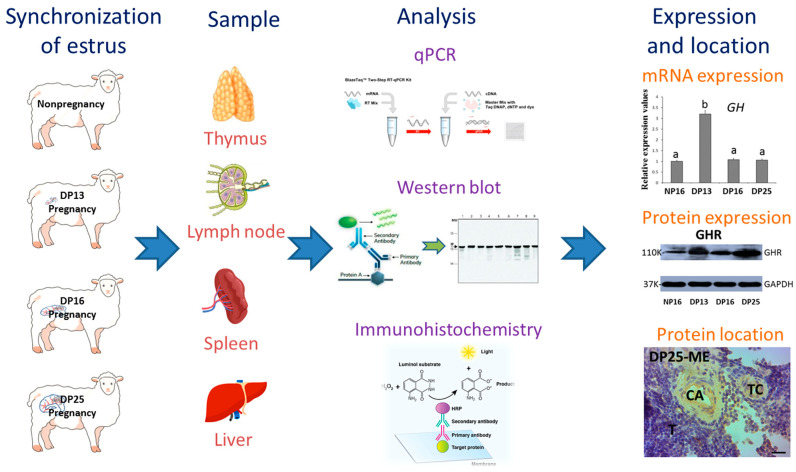
Experimental approach. Different letters indicate significant differences (*p* < 0.05).

The immune organs mainly include the bone marrow, thymus, lymph nodes, spleen, and liver that generate immune cells and/or harbor immune cells to mediate immune responses [13]. In the bovine and ovine, early pregnancy signals (interferon tau, IFNT) and progesterone have effects on maternal immune function during gestation [14]. IFNT exerts effects on maternal immune functions to avoid fetus rejection by the maternal uterus during early gestation [15]. Our previous research shows that IFNT exerts actions on maternal immune organs [16] via blood circulation in ewes. In addition, pregnancy signals modulate expression of melatonin receptors, gonadotropin-releasing hormone and its receptor, prolactin and its receptor, follicle-stimulating hormone and luteinizing hormone receptors, and estrogen receptors in the ovine immune organs [17,18,19], which participate in maternal immune tolerance in a tissue-specific manner. Moreover, early gestation has effects on the expression of interferon-stimulated gene 15 (*ISG15*) mRNA in the ovine anterior pituitary [20]. Therefore, it is supposed that early gestation influences GH and GHR expression in the maternal thymus, lymph node, spleen, and liver. Thus, the objective of this research is to explore GH and GHR expression in these organs from nonpregnant ewes and early gestation females, which will be the first report that reveals the effects of early gestation on modulating the function of these immune organs via GH and GHR (Figure 1). The finding will contribute to revealing the maternal immune tolerance.

## 2. Materials and Methods

### 2.1. Animals and Experimental Design

The experiment was performed in the Hebei Province, China, during normal breeding season (October and November) with an average temperature of 12 °C to 24 °C under a short photoperiod. A total of 24 adult ewes (Small-tail Han sheep, approximately 18 months of age, body condition score of 3) with normal estrus cycles and similar body conditions were randomly divided into four groups and mated with either adult intact rams (pregnant ewes) or a vasectomized ram (nonpregnant ewes) as described previously [17]. The day of estrus onset was assigned as day 0. Maternal thymus, lymph node, spleen, and liver were obtained on days 13, 16, and 25 for pregnant animals (DP13, DP16, and DP25) and on day 16 for nonpregnant ewes (NP16) (*n* = 6 for each group) after euthanasia by an experienced person after electrical stunning. These four different stages were selected according to progesterone and IFNT secretion as described previously [17]. Cross-sections of these organs were prepared for immunohistochemical analysis, and transverse pieces of these organs were frozen in liquid nitrogen for mRNA isolation and protein analyses.

### 2.2. RNA Extraction and RT-qPCR Assay

Total RNA extraction, quantity and quality of total RNA, and reverse transcription to cDNA were performed as described previously (*n* = 6 for each group) [17]. The specified primers are listed in Table 1. Real-time quantitative PCR was performed in a CFX96 real-time PCR detection system (Bio-Rad Laboratories, Hercules, CA, USA), and *GAPDH* was used for normalization of gene expression data. Negative controls (nuclease-free water) and positive controls (cDNA from positive sample control) were included in all assay runs. The 2^−ΔΔCt^ method was utilized to analyze the relative values [21]. The mean Ct values from NP16 were used as reference points, and the Ct values of all the groups were used to calculate the fold change relative to the reference points.

### 2.3. Western Blot Analysis

Western blot analysis was performed as described previously (*n* = 6 for each group) [17]. A mouse anti-GHR antibody (Santa Cruz Biotechnology, Santa Cruz, CA, USA, sc-137185, 1:1000) and a rabbit anti-GH1 antibody (Abcam, Cambridge, UK, ab155974, 1:1000) were used to detect GH and GHR proteins in these tissues. Negative controls without ovine GH and GHR proteins and positive controls with ovine GH or GHR proteins were used to validate the species cross-reactivity of the primary antibodies. A GAPDH antibody (Santa Cruz Biotechnology, sc-47724, 1:1000) was utilized to assess consistent loading. Values are presented as the ratio of GH or GHR-integrated optical density to GAPDH-integrated optical density.

### 2.4. Immunohistochemistry Analysis

Immunohistochemistry was described previously [17]. Some sections were stained by hematoxylin and eosin, and others (*n* = 6 for each group) were incubated with the primary antibody specific to GHR (1:200 dilution; Santa Cruz Biotechnology, sc-137185, Santa Cruz, CA, USA) at 4 °C overnight. For negative control, an antiserum-specific isotype was substituted for the primary antibody at the same protein concentration. A DAB kit (Tiangen Biotech, Beijing, China) was utilized to detect the primary antibody. The images were analyzed by assigning an immunoreactive intensity on a scale of 0 to 3. An intensity of 3 was given to the cells with the highest staining intensity, and an intensity of 0 was assigned to cells with no immunoreactivity as described previously [22].

### 2.5. Statistical Analysis

A completely randomized design using the Proc Mixed models of SAS (version 9.4; SAS Institute Inc., Cary, NC, USA) was performed for statistical analysis, and day and status (nonpregnancy or pregnancy), and day–status interactions were included in this model. The comparisons among the relative expression levels of different groups were made using the Duncan method and controlling the experiment-wise type ± error equal to 0.05. All data obtained from the thymus, lymph node, spleen, and liver were expressed as means ± standard deviation, and *p*-values less than 0.05 were considered to be a significant difference.

## 3. Results

### 3.1. GH and GHR in the Thymus

**Figure 2:**
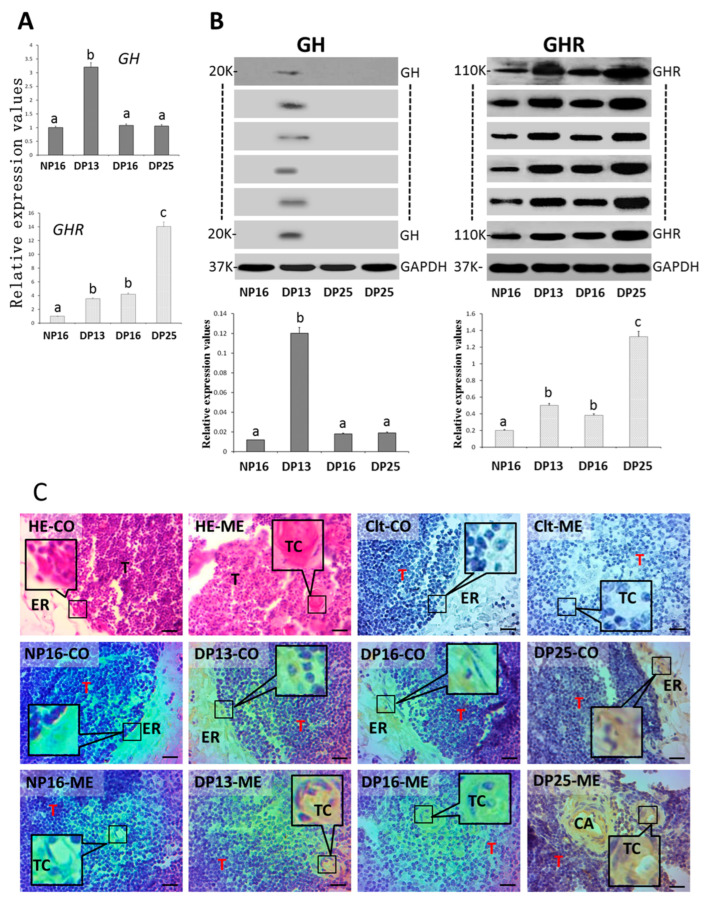
GH and GHR in the thymus. (**A**) Expression values of *GH* and *GHR* mRNA. (**B**) Expression of GH and GHR proteins. (**C**) GHR protein in the thymus. Note: HE = stained by hematoxylin and eosin; Clt = negative control; NP16 = day 16 of nonpregnancy; DP13 = day 13 of pregnancy; DP16 = day 16 of pregnancy; DP25 = day 25 of pregnancy. CO = cortex; ME = medulla; T = thymocyte; ER = epithelial reticular cell; CA = capillary; TC = thymic corpuscle; bar = 20 µm. Different letters indicate significant differences (*p* < 0.05).

Figure 2A,B reveal that gestation improved *GHR* mRNA and protein expression in the thymus (*p* < 0.05), and levels of *GH* mRNA and protein peak at DP13 (*p* < 0.01). Furthermore, GH protein was not detectable at NP16, DP16, and DP25 (Figure 2B and Figure S1). In addition, GHR protein was expressed in the epithelial reticular cells, capillaries, and thymic corpuscles (Figure 2C). The staining intensities for GHR protein were 0, 1, 2, 2, and 3 for the negative control and the samples from NP16, DP13, DP16, and DP25. The staining intensity was as follows: 0 = negative; 1 = weak; 2 = moderate; 3 = strong.

### 3.2. GH and GHR in Lymph Nodes

**Figure 3:**
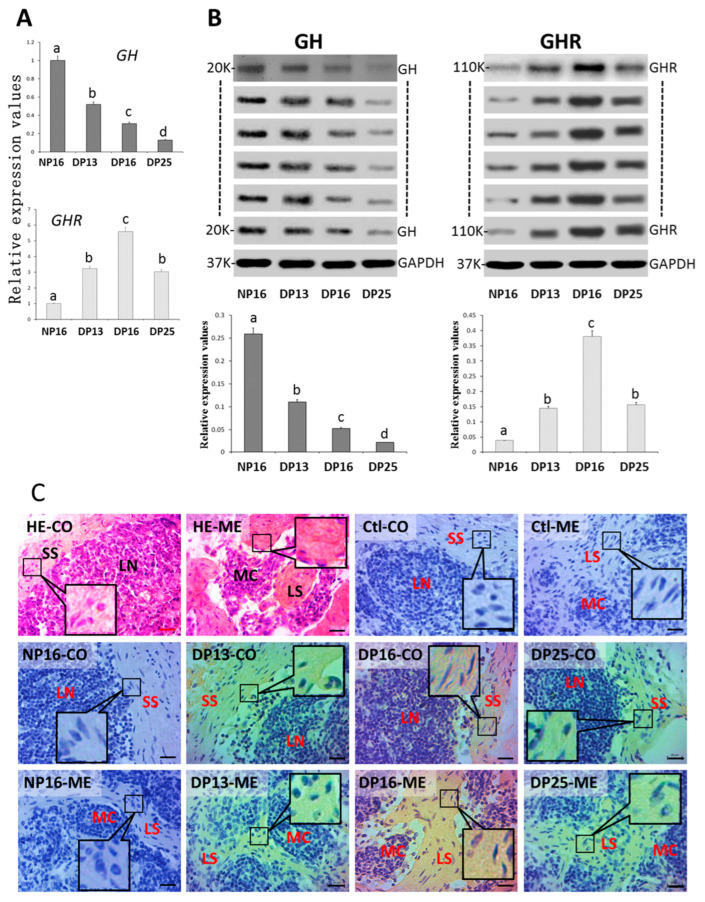
GH and GHR in lymph nodes. (**A**) Expression values of *GH* and *GHR* mRNA. (**B**) Expression of GH and GHR proteins. (**C**) GHR protein in the lymph node. Note: HE = stained by hematoxylin and eosin; Ctl = negative control; NP16 = day 16 of nonpregnancy; DP13 = day 13 of pregnancy; DP16 = day 16 of pregnancy; DP25 = day 25 of pregnancy. CO = cortex; ME = medulla; SS = subcapsular sinus; LN = lymphoid nodules; LS = lymph sinus; MC = medullary cord; bar = 20 µm. Different letters indicate significant differences (*p* < 0.05).

There was a decrease in *GH* mRNA and protein expression values during early pregnancy (Figure 3A,B and Figure S1; *p* < 0.01) compared with the nonpregnant ewes, but *GHR* mRNA and protein levels were upregulated during early pregnancy (*p* > 0.01). *GHR* mRNA and protein levels peaked at DP16. Furthermore, the GHR protein was expressed in the subcapsular sinus and lymph sinus (Figure 3C). The staining intensities for GHR protein were 0, 0, 1, 2, and 1 for the negative control and samples from NP16, DP13, DP16, and DP25.

### 3.3. GH and GHR in the Spleen

**Figure 4:**
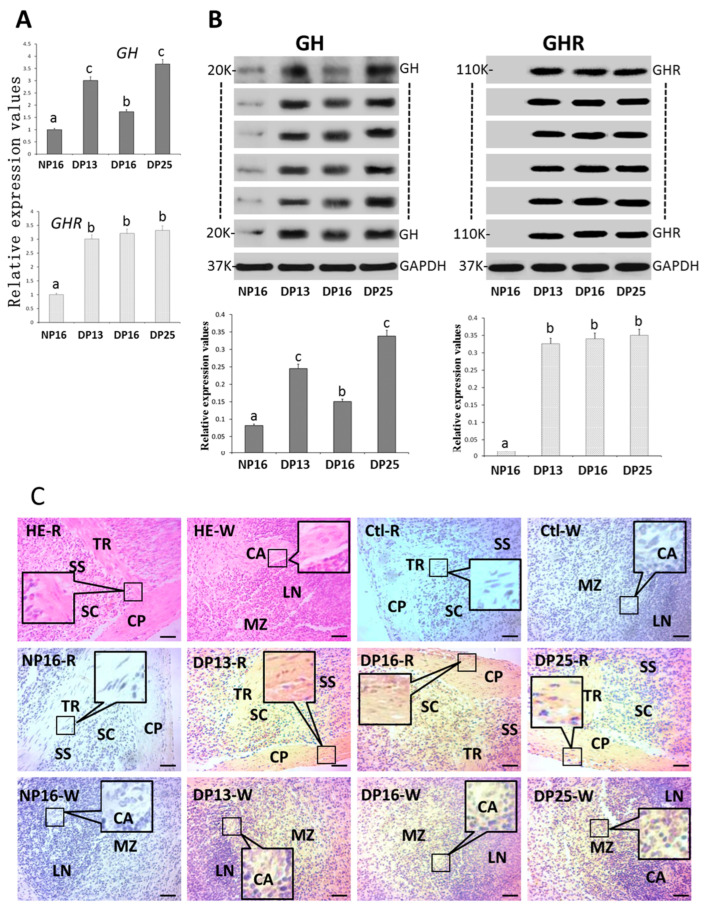
GH and GHR in the spleen. (**A**) Expression values of *GH* and *GHR* mRNA. (**B**) Expression of GH and GHR proteins. (**C**) GHR protein in the spleen. Note: HE = stained by hematoxylin and eosin; NP16 = day 16 of nonpregnancy; DP13 = day 13 of pregnancy; DP16 = day 16 of pregnancy; DP25 = day 25 of pregnancy. R = red pulp; W = white pulp; CP = capsule; TR = trabeculae; Ctl = negative control; SS = splenic sinuses; SC = splenic cords; MZ = marginal zone; LN = lymphoid nodule; CA = central arteriole; bar = 50 µm. Different letters indicate significant differences (*p* < 0.05).

GH and GHR were upregulated in both mRNA and protein levels during early pregnancy compared with NP16 (*p* < 0.05). The GHR protein was not detectable at NP16 (Figure 4B and Figure S1). GHR protein was expressed in the capsule, trabeculae, splenic cords, and marginal zone (Figure 4C). The staining intensities for GHR protein were 0, 0, 2, 2, and 2 for the negative control and the samples from NP16, DP13, DP16, and DP25.

### 3.4. GH and GHR in the Liver

**Figure 5:**
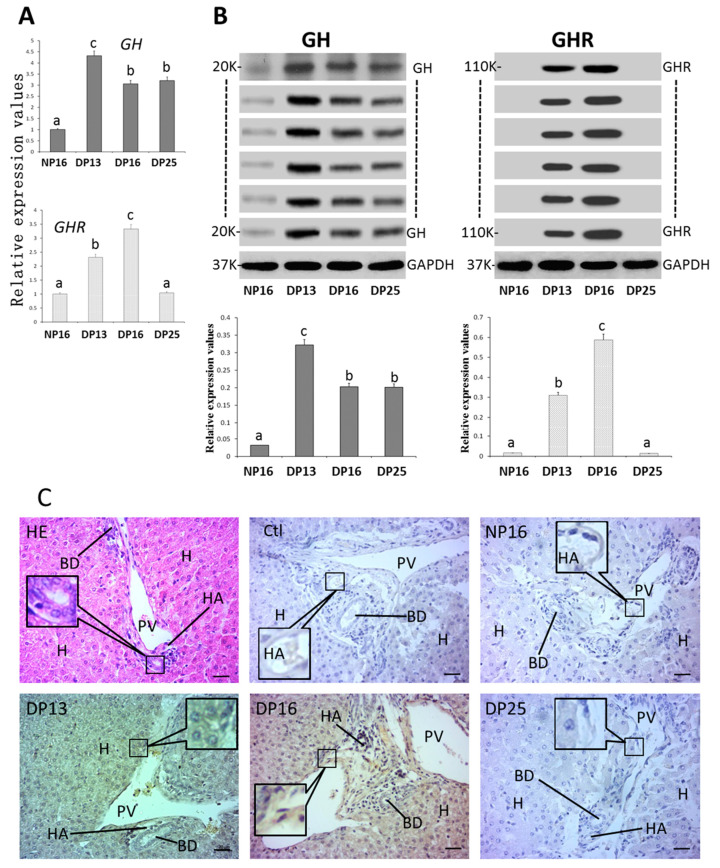
GH and GHR in the liver. (**A**) Expression values of *GH* and *GHR* mRNA. (**B**) Expression of GH and GHR proteins. (**C**) GHR protein in the liver. Note: HE = stained by hematoxylin and eosin; Ctl = negative control; H = hepatocyte; NP16 = day 16 of nonpregnancy; DP13 = day 13 of pregnancy; DP16 = day 16 of pregnancy; DP25 = day 25 of pregnancy. HA = hepatic artery; PV = portal vein; BD = bile duct; bar = 50 µm. Different letters indicate significant differences (*p* < 0.05).

Expression levels of *GH* mRNA and protein were enhanced during early gestation compared with NP16 (Figure 5A,B and Figure S1; *p* < 0.01). Nevertheless, values of *GHR* mRNA and protein were high at DP13 and DP16 compared with NP16 and DP25 (*p* < 0.01), and GHR proteins were not detected at NP16 and DP25. GHR protein was expressed in the hepatocytes and endothelial cells of the proper hepatic arteries and hepatic portal veins (Figure 5C), and the staining intensities for GHR were 0, 0, 2, 2, and 0 for the negative control and samples from NP16, DP13, DP16, and DP25.

## 4. Discussion

Resident B cells and non-epithelial perivascular spaces in the thymus are related to central tolerance [23]. There are thymic involution and expansion of thymic natural regulatory T cells in pregnant female mice, which are related to progesterone and osteoclast differentiation receptors [9]. GH increases the proliferation of thymocytes and thymic epithelial cells and induces secretion of thymic hormones, which results in increases in thymocyte migratory responses [24]. Administration of GH enhances DNA synthesis in the thymus and recovers the immune response, and GH has beneficial effects on the regeneration of the thymus in rats [25]. The thymic stromal cells express GH and result in higher local concentrations of GH than systemic ones, which regulate the thymic microenvironment for T-lymphocyte differentiation in humans [26]. Human thymic cells produce GH, and thymic epithelial cells and thymocytes express GHR, which modulate thymic functions in an autocrine/paracrine manner [27]. Increases in placental GH during pregnancy gradually take the place of pituitary GH [28]. Our results indicated that GH mRNA and protein increased only on DP13, but GHR was upregulated during early gestation, and the GHR protein was expressed in the epithelial reticular cells, capillaries, and thymic corpuscles. Thymic corpuscles represent a subset of thymic epithelial cells that influence T-cell development [29]. Thus, GH may modulate thymic functions in an autocrine/paracrine and endocrine manner via GHR during early pregnancy.

Lymph enters and exits lymph nodes and eventually returns to the blood circulation, which is involved in modulating immune responses [30]. Leukocytes enter lymph nodes via the sinus system that coordinates immune responses in humans [31]. The plasma GH level correlates with submaxillary lymph node immune responses and lymphocyte subset populations in male rats [32]. There is a higher level of circulating GH during pregnancy compared with nonpregnancy in humans, which is not related to GH-releasing hormone [33]. GH modulates lymphocyte migration of the immune system, including lymph nodes, in GH transgenic mice [34]. GHR expressed in the immune system regulates its function via endocrine, paracrine, and autocrine mechanisms [35]. Our results indicated that the GH relative expression level was downregulated, but GHR was upregulated during early gestation in the female lymph node, and GHR protein was expressed in the subcapsular sinuses and lymph sinuses. It is known that placental GH is upregulated during pregnancy [28]. Therefore, the upregulation of GHR may be related to the placental GH, which is associated with reconstruction of the function of the maternal lymph system in autocrine, paracrine, and endocrine manners during early pregnancy.

The intricate positioning of the immune cells within the spleen and the ways of their migration are involved in the regulation of adaptive immunity in the spleen [36]. GH can regulate expression of cytochrome P450 2C11 in the spleen, which is involved in catalyzing a large variety of essential metabolites in rats [37]. Injection of GH significantly improves the function of human hematopoietic stem cells and immune cell reconstitution in the spleen [38]. GH injection also increases circulating GH concentrations and stimulates expression of *GHR* mRNA in the spleen of rats [39]. Synthesis of lymphocyte GH increases in the rat spleen, which is induced by hypoxia and cytoplasmic alkalinization [40]. The spleen produces and secretes GH that can stimulate the cytotoxic activity of natural killer cells and induce lymphocyte proliferation through binding GHR [41]. Our finding indicates that GH and GHR expression was enhanced, and the GHR protein was expressed in the capsule, trabeculae, and splenic cords. Thus, the increases in GH and GHR may modulate the female splenic function in a paracrine and autocrine manner during early gestation.

The liver contains diverse immune cells and works as a lymphoid organ to facilitate maternal immune tolerance during pregnancy [5]. GH modulates metabolic, immune, and hepatic stellate cell function, which is related to hepatic steatosis, inflammation, and fibrosis [42]. GH can activate autophagy through GHR in the liver of the chronically starved mice [43]. GH also regulates adult metabolism to protect against the development of steatosis, which is via hepatic GHR signaling [44]. Decrease in somatostatin secretion from the hypothalamus and increase in expression level of the *GH* gene in the pituitary enhance plasma GH levels, but GHR mRNA progressively decreases in the liver during pregnancy in rats [45]. During pregnancy, the upregulation of placental GH and pituitary GH improves maternal insulin-like growth factor 1 levels, which modulates glucose homeostasis in the liver [46]. Our finding indicated that GH expression increased during early gestation, but GHR was downregulated on DP25. Thus, the increase in GH and the altered expression of GHR may contribute to the regulation of hepatic immune tolerance in an endocrine and paracrine/autocrine manner during pregnancy.

## 5. Conclusions

Early pregnancy modulates GH and GHR expression in the maternal thymus, lymph nodes, spleen, and liver in a tissue-specific style, which may be via an endocrine and paracrine/autocrine manner. Therefore, it is suggested that the modulation of the expression of GH and GHR in the maternal thymus, lymph nodes, spleen, and liver may be involved in regulating the function of these immune organs during early gestation. In addition, IFNT and progesterone, as well as pituitary GH, may be involved in regulating the expression of GH and GHR. However, the functional significance of GHR localization in specific structures of immune organs and potential influences, including feed, season, or the other animal model, should be further explored.

## Figures and Tables

**Table 1:** Primers used for RT-qPCR.

Gene	Primer	Sequence	Size (bp)	Accession Numbers
*GH*	Forward	GCAGTTCCTCAGCAGAGTCTTCAC	90	NM_001009315.3
	Reverse	ATGCCTTCCTCCAGGTCCTTCAG		
*GHR*	Forward	CAGTGTGACACGCACCCAGAAG	84	NM_001009323.2
	Reverse	GGCATCTACCTCGCAGAAGTAAGC		
*GAPDH*	Forward	GGGTCATCATCTCTGCACCT	176	NM_001190390.1
	Reverse	GGTCATAAGTCCCTCCACGA		

## Data Availability

Data supporting the findings of this study are available within this paper.

## Supplementary Materials

The following supporting information can be downloaded at: https://www.mdpi.com/article/10.3390/biology14101318/s1, Figure S1: Original Western blot.

## Abbreviations

GH	Growth hormone
GHR	Growth hormone receptor
IFNT	Interferon tau
ISG15	Interferon-stimulated gene 15
CO	Cortex
ME	Medulla
ER	Epithelial reticular
CA	Capillary
TC	Thymic corpuscle
SS	Subcapsular sinus
LN	Lymphoid nodule
LS	Lymph sinus
MC	Medullary cord
CP	Capsule
TR	Trabeculae
SC	Splenic cords
MZ	Marginal zone
HA	Hepatic artery
PV	Hepatic portal vein
BD	Bile duct
