# Supplementary material for: Gestation Regulates Growth Hormone and Its Receptor Expression in Sheep Immune Organs
Source: Biology (Basel). 2025 Sep 24;14(10):1318. doi: 10.3390/biology14101318 (PMC12561092; doi:10.3390/biology14101318)

Figure S1 Original Western Blot

WB full membrane for Figure 2

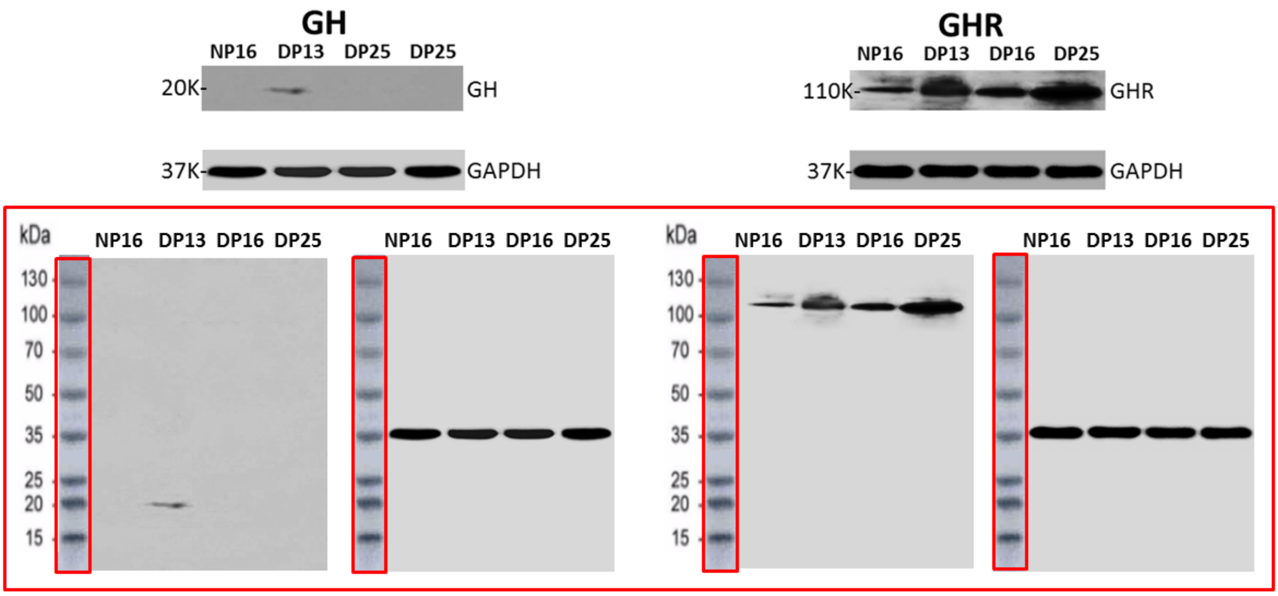

WB full membrane for Figure 3

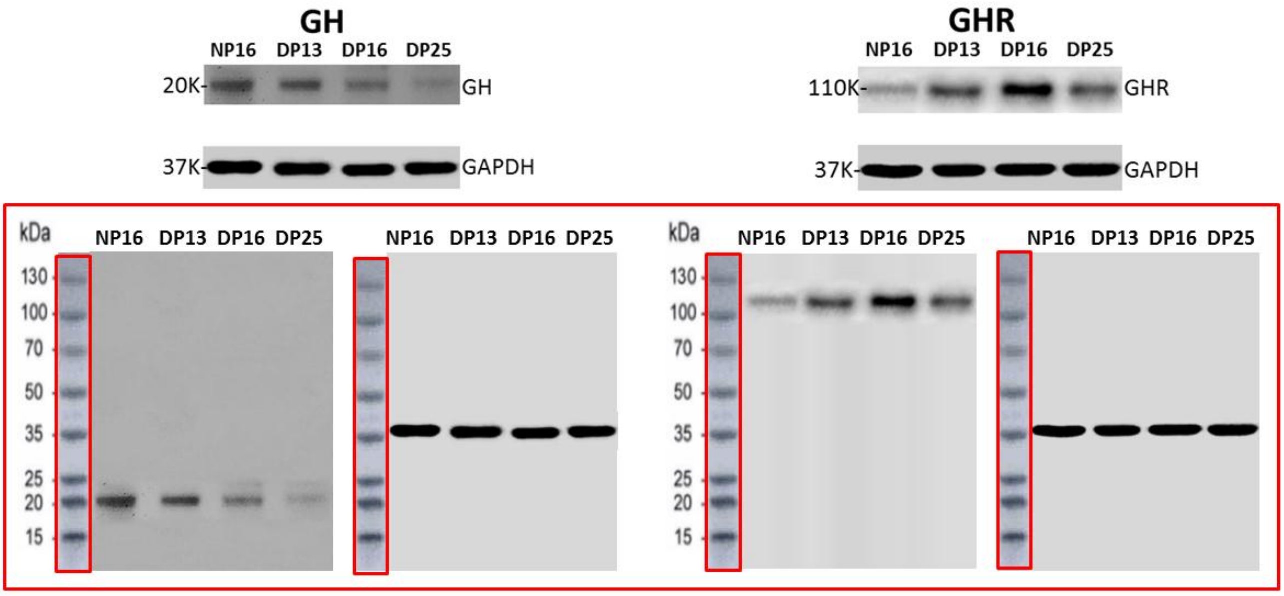

WB full membrane for Figure 4

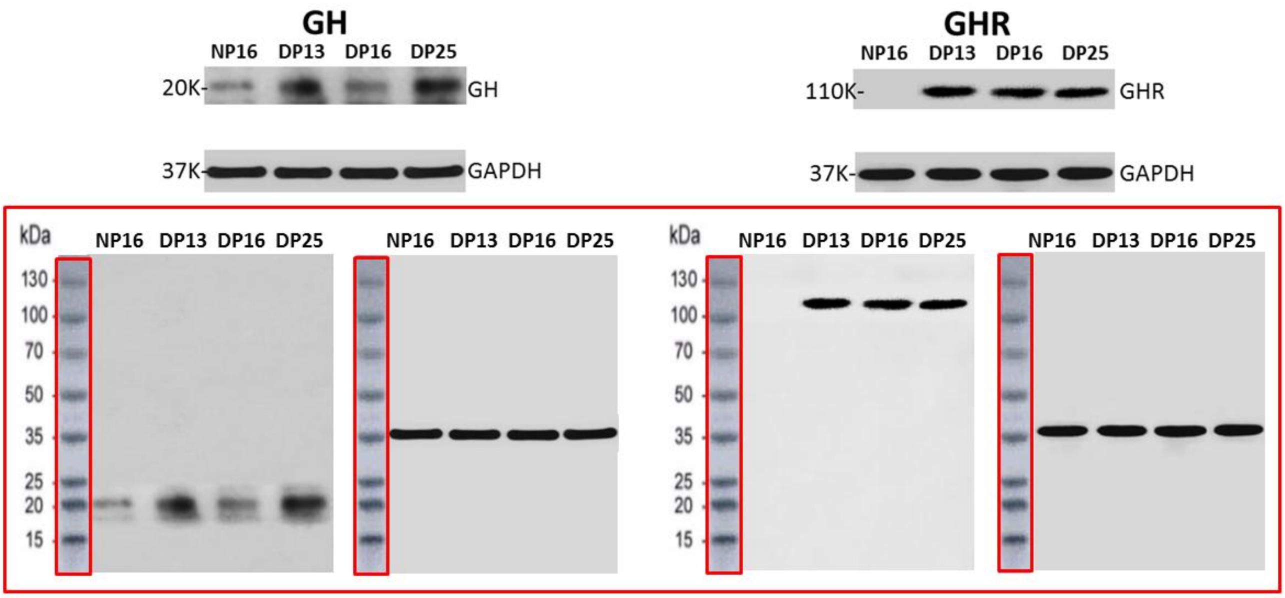

WB full membrane for Figure 5

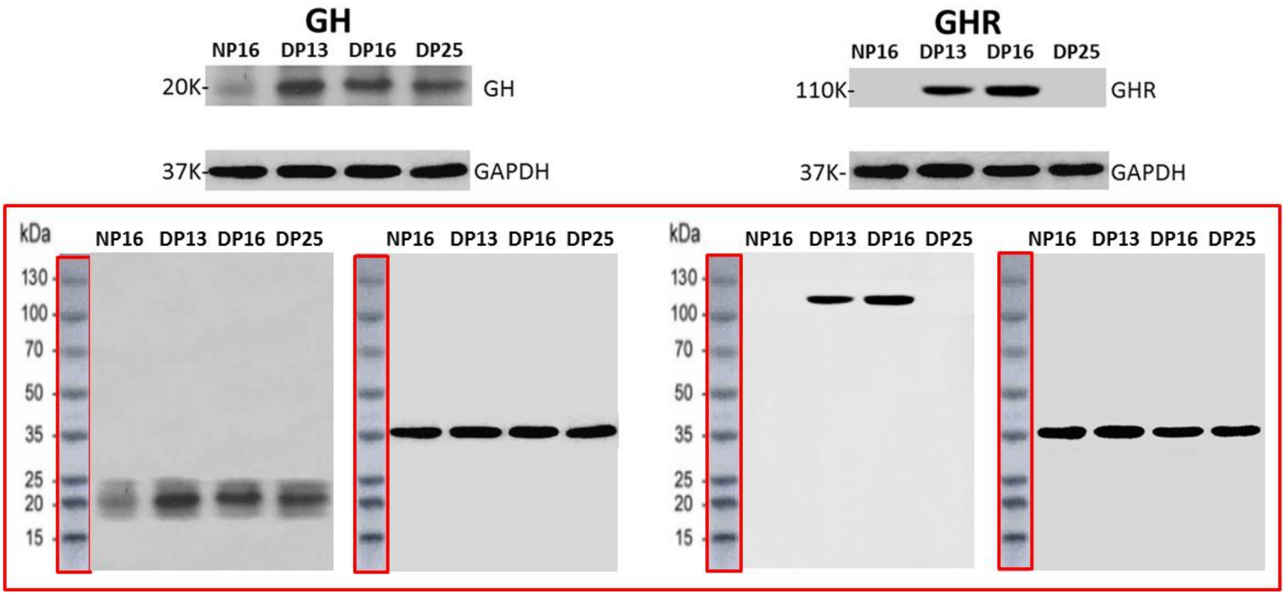

Supplement: Supplementary file 1 [file biology-14-01318-s001.zip › biology-3803593-SI.pdf]
